# Underwater Multi-Channel MAC with Cognitive Acoustics for Distributed Underwater Acoustic Networks

**DOI:** 10.3390/s24103027

**Published:** 2024-05-10

**Authors:** Changho Yun

**Affiliations:** Korea Research Institute of Ships & Ocean Engineering (KRISO), Daejeon 34103, Republic of Korea; sgn0178@kriso.re.kr; Tel.: +82-42-866-3834

**Keywords:** acoustic frequency band, cognitive acoustic, distributed topology, interference, medium access control, underwater cognitive acoustic network

## Abstract

The advancement of underwater cognitive acoustic network (UCAN) technology aims to improve spectral efficiency and ensure coexistence with the underwater ecosystem. As the demand for short-term underwater applications operated under distributed topologies, like autonomous underwater vehicle cluster operations, continues to grow, this paper presents Underwater Multi-channel Medium Access Control with Cognitive Acoustics (UMMAC-CA) as a suitable channel access protocol for distributed UCANs. UMMAC-CA operates on a per-frame basis, similar to the Multi-channel Medium Access Control with Cognitive Radios (MMAC-CR) designed for distributed cognitive radio networks, but with notable differences. It employs a pre-determined data transmission matrix to allow all nodes to access the channel without contention, thus reducing the channel access overhead. In addition, to mitigate the communication failures caused by randomly occurring interferers, UMMAC-CA allocates at least 50% of frame time for interferer sensing. This is possible because of the fixed data transmission scheduling, which allows other nodes to sense for interferers simultaneously while a specific node is transmitting data. Simulation results demonstrate that UMMAC-CA outperforms MMAC-CR across various metrics, including those of the sensing time rate, controlling time rate, and throughput. In addition, except for in the case where the data transmission time coefficient equals 1, the message overhead performance of UMMAC-CA is also superior to that of MMAC-CR. These results underscore the suitability of UMMAC-CA for use in challenging underwater applications requiring multi-channel cognitive communication within a distributed network architecture.

## 1. Introduction

Underwater acoustic communication stands out as a reliable wireless technology for transmitting data over extensive distances, ranging from hundreds of meters to tens of kilometers [1,2]. This advantage makes it indispensable for a variety of underwater applications including scientific observation, the exploitation of ocean resources, disaster detection, military surveillance, leisure activities, and subsea construction [3,4].

The acoustic frequency band used for communication, spanning from hundreds of hertz for long-distance data transmission to hundreds of kilohertz for short-distance data transmission, is an open spectrum [5,6]. This implies that any data transmission in the acoustic frequency band may encounter collisions with diverse interferers such as sonar devices, vessel noises, or underwater mammals [7,8]. Currently, there is also an increasing need to develop eco-friendly underwater acoustic communication systems that minimize harm to the underwater ecosystem including harm to divers, coral reefs, and dolphins [9,10]. To address the challenge of a degraded spectral efficiency resulting from frequent interferences and to ensure harmonious coexistence with the underwater ecosystem, there is a notable shift from current underwater sensor networks towards the adoption of underwater cognitive acoustic networks (UCANs) applying Cognitive Acoustic (CA) technology [11,12].

The technologies of UCANs present challenges due to their apparent differences from cognitive radio networks (CRNs). While CRNs benefit from well-defined channel models and standardized licensing policies, UCANs encounter complexities in predicting channel behavior and contend with unpredictable interferers in the open spectrum nature of the acoustic frequency band. Unlike CRNs, which adhere to a framework of primary and secondary users, users with applied CAs are referred to as Cognitive Users (CUs), while interferers without CAs are classified as Non-Cognitive Users (NCUs) in UCANs [13]. Due to the absence of a licensing policy, avoiding interferers that are similar to Primary Users (PUs) in CRNs is necessary to minimize collisions and utilize the narrowband acoustic frequency band more efficiently. Furthermore, while CRNs employ standardized signaling formats for reliable signal decoding, UCANs often face incomprehensible signals from neighboring NCUs, complicating signal interpretation.

Nowadays, there is also a prevalent use of short-term underwater applications employing underwater nodes such as autonomous underwater vehicles [4,14]. These applications are often characterized by operational durations of less than a day, on-demand requirements, and suitability for a self-organized and distributed network architecture. However, these underwater nodes, whether operating wirelessly or tethered, are susceptible to loss due to severe underwater environments (e.g., waves, currents, or winds). Therefore, to ensure the accurate tracking of their status and location, it is imperative to provide periodic data transmission opportunities without collisions with interferers.

Considering these applications, this paper addresses a UCAN with a distributed topology. Specifically, we concentrate on tackling an appropriate medium access control (MAC) protocol for a distributed UCAN. In this protocol, CUs autonomously sense NCUs and utilize the sensing results to select frequencies in a distributed manner. In addition, MAC protocols for a distributed UCAN need to consider several key requirements to deal with vivid communication failures under harsh underwater environments. These include minimizing the message overhead for sharing sensing information, ensuring the sensing time aligns with the data transmission time to accurately investigate the channel occupancy of neighboring NCUs, and offering periodic data transmission opportunities to monitor CU activity and survival statuses.

In the literature, UCAN technologies primarily focus on the efficient allocation of resources such as frequency (or channel), power, or data rate heuristically or optimally [15,16,17,18,19]. However, research on resource allocation or channel access for CUs in a distributed topology has been limited. For instance, in [20], a dynamic control channel MAC is designed, which adaptively adjusts the bandwidth used for control by CUs based on their traffic for a distributed acoustic network. Another approach, discussed in [21], entails that a sender CU in a decentralized network transmits a message containing sensing information to a receiver CU via control channels. Then, the receiver CU selects the data channel that maximizes the channel-sharing reward and finishes channel reservation. Additionally, in [22], a resource allocation method is designed considering the traffic characteristics of neighboring sender CUs. Based on traffic conditions, the receiver CU allocates the sender CUs into a pair of channel and transmission power to maximize their transmission rate.

Previous studies have highlighted the common drawback of increased message overhead due to frequent channel reservation attempts in poor channel conditions, leading to decreased network throughput. This can result in failing to meet the requirement of a distributed UCAN. Accordingly, a new MAC protocol specifically tailored for a distributed UCAN needs to be developed. Most underwater network technologies, notably MAC and routing, have been evolved from terrestrial wireless communication network technologies adapted to the challenging underwater environment [23,24]. Similarly, we aim to design a MAC protocol for distributed UCANs based on well-known distributed MAC protocols developed for terrestrial CRNs. To achieve this, we draw upon the design framework of the Multichannel MAC-Cognitive Radio (MMAC-CR) protocol, as outlined in [25].

MMAC-CR, primarily designed for cognitive radio (CR) technology, exploits idle frequency bands during non-viewing periods of TV viewers, who function as NCUs. However, directly applying MMAC-CR to a UCAN may pose several challenges due to the distinct differences between the environment of MMAC-CR and that of a distributed UCAN. These challenges include an insufficient sensing time relative to the total frame duration, a longer duration allocated for sensing and channel selection compared to the data transmission time, leading to frequent time delays in underwater environments with long propagation delays. Additionally, the increased collision rates caused by NCUs, along with the significant signaling overhead before data transmission, can potentially decrease network throughput. Moreover, there is a lack of assurance for consistent channel access in UCANs when utilizing MMAC-CR.

In this paper, we propose an Underwater Multichannel MAC-Cognitive Acoustic (UMMAC-CA) protocol designed for a distributed UCAN. In UMMAC-CA, we seek to address the challenges posed by the long propagation delays of underwater acoustic signals and the random occurrence of NCUs by reducing the interval between sensing and data transmission. This is because not only the sensing duration but also the timing of sensing is important. Given the likelihood of frequent underwater communication failures and the distributed nature of the environment, there is a risk of not receiving or generating control messages, leading to irregularities in message transmission and updates within the network. Such disruptions could render the entire network inoperable for extended periods, significantly impacting spectrum efficiency and throughput. Hence, we advocate for a simple and regular channel access approach to mitigate potential damage during unfavorable channel conditions in UMMAC-CA. Then, UMMAC-CA is characterized as follows:

- To minimize the time between sensing and data transmission, a single frame is divided into multiple subframes. Each subframe sequentially conducts channel scanning, channel selection based on sensing information, and data transmission;
- Only one CU transmits data in each subframe to prevent collisions and back-off time during simultaneous data transmission attempts by multiple CUs. Although this method restricts the parallel use of all available channels, it affords more data communication opportunities for all CUs compared to the frame structure of MMAC-CR. While MMAC-CR allows data transmission only on the interference-free channels per frame, UMMAC-CA offers increased data communication chances based on the number of subframe repetitions within a frame;
- CUs maintain a pre-determined transmission schedule to autonomously determine the data transmission order. Leveraging this schedule, CUs independently decide on data transmission based on frame and subframe index values. This mechanism ensures that if the sender CU in a specific subframe fails to transmit data to the sender CU, this does not impact the operation of other CUs in the subsequent subframe. Therefore, the use of a pre-determined data transmission matrix not only eliminates interference with other CUs but also reduces the message overhead associated with channel occupancy;
- During the data exchange among specific sender and receiver CUs within a subframe, CUs that are neither transmitting nor receiving data sense the status of NCUs. This approach enables the sender or receiver CU of the next subframe to select channels for data transmission or reception without the need for additional NCU sensing time;
- In MMAC-CR, all CUs sense the data channels in Phase I, share sensing information in Phase II, occupy channels in Phase III, and perform data transmission in Phase IV. In contrast, UMMAC-CA utilizes the pre-determined data transmission matrix in each subframe, allowing only a specific sender–receiver CU pair to reserve channels and exchange data per subframe, while the rest of the CUs sense the data channels. Particularly, since sensing information is not shared across the network as it is in MMAC-CR, there is no associated control signal overhead. Therefore, even though the frame is fragmented into subframes, the working load of each CU does not significantly increase. However, CUs not involved in data transmission in a subframe need to sense the data channels. This may lead to increase the received power consumption for sensing, which is proportional to the number of subframes.

Due to the above characteristics, UMMAC-CA can meet the requirements of reducing message overhead, ensuring sensing time is equal to data transmission time, and providing periodic channel access opportunities in a distributed UCAN. In particular, the sensing of NCUs by CUs not involved in data transmission during the data transmission of specific CUs guarantees a consistent ratio of sensing time to the total frame time. This enables more agile responses to randomly occurring NCUs, reducing the probability of collisions with NCUs and enhancing network throughput.

The remainder of this paper is structured as follows. Section 2 assesses the feasibility of MMAC-CR for distributed UCANs. Section 3 explains UMMAC-CA in detail. In Section 4, the performance of UMMAC-CA is analyzed via simulations. Finally, the conclusions are described in Section 5.

## 2. Feasibility Analysis of MMAC-CR for UCANs

**Figure 1:**
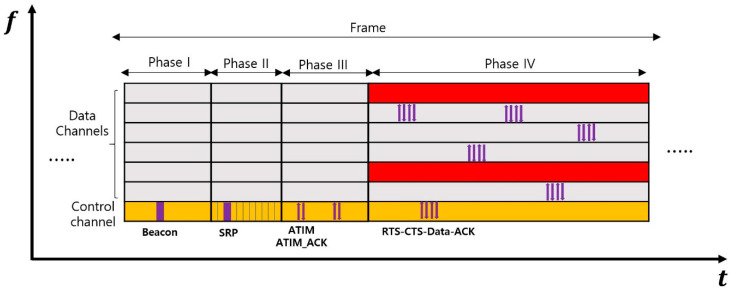
The frame structure of MMAC-CR [25].

Figure 1 illustrates the frame structure of MMAC-CR as shown in [25]. A single frame consists of four phases, and each phase is outlined as follows:

- Phase I is the time during which nodes synchronize their frames and scan the availability of data channels. To accomplish this, nodes competitively broadcast beacons;
- Phase II is the time dedicated to sharing the sensing results of data channels among the nodes in the network, and this consists of mini-slots corresponding to the number of data channels. In each mini-slot, nodes transmit data channel status information based on the Distributed Coordination Function (DCF). If a node receives a Scan Result Packet (SRP) during that slot, it backs off for a certain period;
- Phase III is the time during which nodes utilize the sensing results of data channels to reserve data channels for data transmission between sending and receiving nodes on available data channels. This process involves the use of an Ad hoc Traffic Indication Message (ATIM) and ATIM_ACKnowledgement (ATIM_ACK) messages;
- Phase IV is the period during which nodes engage in data transmission and reception based on the channel reservation results, facilitated by the exchange of Request To Send (RTS)-Clear To Send (CTS)-Data-ACK messages between two nodes. The blocks highlighted in red in Phase IV of Figure 1 represent unavailable data channels. Nodes can transmit and receive data on the remaining data channels excluding these channels.

The propagation delay of acoustic signals for transmitting 1.5 km away is approximately one second [26]. To present the length of each phase, we define $\tau_{p}$, $\tau_{d}$, and $\tau_{g}$ as the maximum propagation delay, the transmission delay, and the guard time, respectively. Also, C and N are, individually, the number of data channels and the number of CUs. When applying MMAC-CR directly to a UCAN with such considerable propagation delays of acoustic signals, the estimated time for each phase can be found as follows:

- The length of Phase I is defined as $\tau_{p}+\tau_{d}+\tau_{g}$ by assuming the beacon is propagated to the CUs at the network edge. $\tau_{p}+\tau_{d}+\tau_{g}$ is represented as τ;
- Since Phase II consists of multiple slots equal to the number of data channels, the length of Phase II is expressed as C×τ. In [25], although the slot length was initially provided as being very short, it must be adjusted to account for the maximum propagation delay required for an SRP message to reach the CUs at the network edge in an underwater environment;
- The length of Phase III is derived considering a simple scenario where all CUs are fully occupied to account of the most complex scenario; all CUs transmit and receive ATIM-ATIM_ACK messages sequentially. The length of Phase III is determined as 2×N×τ under the assumption that each ATIM-ATIM_ACK message exchange per CU can be completed without collisions within 2×τ;
- In [25], multiple CUs attempt data transmission on idle data channels during Phase IV. However, this concept is inefficient for a UCAN due to the potential increases in message overhead and power consumption caused by back-off mechanisms in case of unfavorable channel conditions or collisions. Therefore, this paper assumes that only one CU occupies an idle data channel and performs data transmission to avoid cascading collisions and delays. Since the sender CU and receiver CU exchange four messages (RTS-CTS-Data-ACK), the length of Phase IV is represented as 4×τ;
- The length of a frame is finally expressed as $\left(2N+C+5\right)\times \tau$. In a UCAN, due to the limited bandwidth and low propagation velocity, the propagation delay is generally greater than the transmission delay. Moreover, when the communication distance is several kilometers, the propagation delay is typically on the order of seconds. Therefore, depending on the number of data channels and CUs, the length of a frame can range from tens of seconds to minutes.

Through the analysis of the length of each phase, the limitations of applying MMAC-CR to a UCAN can be summarized as follows:

- To prevent collisions caused by NCUs, the frequent sensing of their status is necessary. However, applying MMAC-CR directly to a UCAN may lead to Phase I being too short for adequate sensing, resulting in an inability to adapt to changes in the NCU status during subsequent phases. This timing mismatch between sensing and sharing channel occupancy information can significantly increase the likelihood of NCU-induced collisions in Phase IV;
- Additionally, Phases II and III, focused on sharing the channel state and selecting data channels, are considerably longer than Phase IV, where data transmission occurs. This disparity in duration may elevate the collision rates due to NCUs and prolong the time needed for control signaling, ultimately reducing network throughput. Consequently, the proportion of time allocated to actual data transmission within the total frame time diminishes;
- Thus, it is necessary to reduce frame length through efficient channel state sharing, channel occupancy, and data transmission. This involves developing more effective methods for sharing channel state and occupancy while minimizing the number of control messages. Moreover, alternative channel access methods, less prone to time delays like those encountered with 802.11 DCF back-offs, should be explored for a distributed UCAN.

## 3. UMMAC-CA

In Section 3, we explain UMMAC-CA for a distributed UCAN, including the network model, frame definition, transmission scheduling, and data transmission method. The parameters used in UMMAC-CA are summarized in Table 1.

### 3.1. Network Model

**Figure 2:**
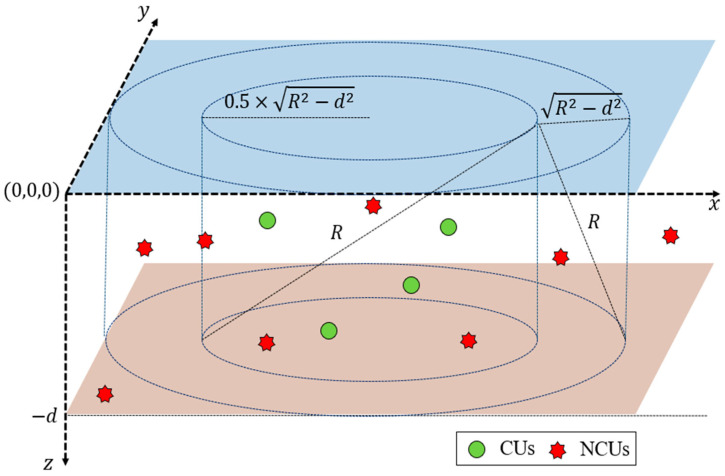
The configuration and topology of a distributed UCAN.

We consider a three-dimensional distributed UCAN, as depicted in Figure 2. The network is composed of multiple CUs and NCUs. In Figure 2, the blue area implies the water surface, and the brown area means the sea-bed. To carry out CAs, CUs have communication modules to sense the overall underwater acoustic frequency band and choose a desired acoustic frequency.

CUs are located in a cylinder with a radius of $0.5\times \sqrt{R^{2}-d^{2}}$ and a height of d (i.e., the inner cylinder in Figure 2). CUs exist in a location where one-hop communication is possible within the maximum communication range (R). The location of a CU is represented in x-y-z coordinates and the maximum values of the x and y axes are expressed as R and d. The x and y coordinates of a CU are randomly set in the range of $\left[\sqrt{R^{2}-d^{2}},2\times \sqrt{R^{2}-d^{2}}\right]$, and the z coordinate of a CU is arbitrarily determined in the range of $\left[0,-d\right]$. CUs can move within the network range.

Only NCUs within an area with twice the maximum communication range can be sensed, so we consider NCUs located in a cylinder with a radius of $1.5\times \sqrt{R^{2}-d^{2}}$ and a height of d (i.e., the outer cylinder in Figure 2). In this area, NCUs randomly occur in both the time and frequency domains. As a result, the x and y coordinates of an NCU are randomly set in the range of $\left[0,3\times \sqrt{R^{2}-d^{2}}\right]$, and the z coordinate of an NCU is randomly set in the range of $\left[0,-d\right]$.

The activity of an NCU is modelled in terms of the number of occurring NCUs ($N_{NCU}$) per data channel, the occurrence time of each NCU ($t_{NCU}$), and the occurrence time duration of each NCU ($N_{NCU}$), as described in [13]. $t_{NCU}$ and $T_{NCU}$ are modeled to have a uniform distribution in the range of $\left[\left(FI-1\right)\times T,FI\times T\right]$ and $\left[1,\;T_{MAX}\right]$, respectively, where $T_{MAX}\;$ is the maximum occurrence time duration. $N_{NCU}$ is modelled to have a Poisson distribution with an average of $\lambda_{NCU}$.

As there are no central entities that control the network overall, CUs’ nodes exist freely within the network range, occupying data channels to transmit data with each other, thus forming a distributed network structure. CUs autonomously sense NCUs and, based on their own sensing information, determine a data channel between specific sender and receiver CUs. To achieve this, CUs adhere to the same time frame structure, performing sensing, channel negotiation, and data transmission accordingly within this frame.

### 3.2. Division in the Time and Frequency Domains

**Figure 3:**
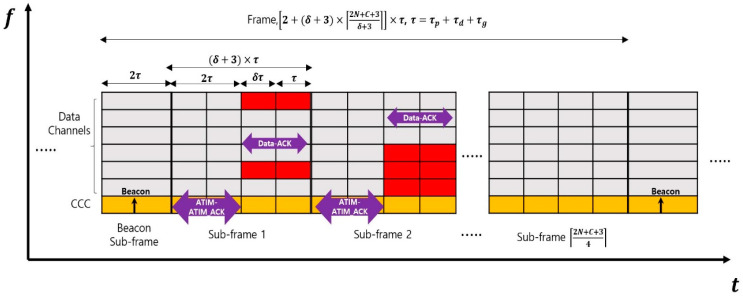
The frame structure of UMMAC-CA.

To maintain consistency with MMAC-CR, we consider the frequency domain to comprise one control channel and C data channels in the frequency domain. In the time domain, however, the frame structure of UMMAC-CA consists of multiple subframes, as depicted in Figure 3. The Beacon subframe is the first subframe in one frame, and it is dedicated to broadcasting the beacon, akin to MMAC-CR. Subsequent subframes facilitate data channel selection using sensing information and the data transmission between sender and receiver CUs.

The Beacon subframe serves as a time for network synchronization, broadcasting beacons similar to MMAC-CR, and transmitting the data transmission order matrix of CUs, while also sensing the statuses of NCUs on the data channels. To prevent collisions, only a subset of CUs in the network may broadcast beacons per frames. Broadcasting a beacon by a single CU could risk loss due to channel contention, so network CUs are grouped, with each group randomly broadcasting beacons. Moreover, to reduce collision probability, the length of this subframe is set to 2τ, allowing CU groups to broadcast beacons at random intervals within twice the maximum propagation delay time.

The remaining subframes, excluding the Beacon subframe, are indexed from 1 to x. These subframes are dedicated to the process where a specific sender CU determines the data channel for data transmission with a receiver CU via the exchange of ATIM-ATIM_ACK messages and conducts data transmission through the exchange of Data-ACK messages. In Figure 3, the red colored area implies the available data channels for the sender and receiver CUs. The length of these subframes can be represented as $\left(2+2\delta \right)\times \tau$, considering the maximum propagation delay for both uplink and downlink. This is because the time for exchanging ATIM-ATIM_ACK messages and Data-ACK messages, as seen in Figure 2, becomes 2τ and 2δ×τ, respectively. In this case, δ is greater than 1, indicating that more time is allocated for data transmission compared to sensing time to reduce overhead and increase throughput.

To design UMMAC-CA under the same conditions as MMAC-CR, the length of a frame in UMMAC-CA is set to be equal to the frame length of MMAC-CR. By leveraging this approach, we can derive the number of subframes x within one frame as $\left\lceil \frac{2N+C+3}{\delta +3}\right\rceil$, and consequently, determine the frame length of UMMAC-CA as $T=\left[2+\left(\delta +3\right)\times \left\lceil \frac{2N+C+3}{\delta +3}\right\rceil \right]\times \tau$.

### 3.3. Transmission Scheduling

This section describes the method of configuring sender CUs to transmit data in a specific set of a frame and a subframe. Unlike in CRNs where there are many concurrent users accessing channels with significant randomness in their locations, UCANs primarily utilize a limited number of CUs in an on-demand fashion. Therefore, the emphasis is on providing fixed transmission opportunities to CUs, considering the on-demand nature of their utilization in the underwater environment. This approach also reduces the overhead and power consumption associated with channel access in a distributed network structure.

**Figure 4:**
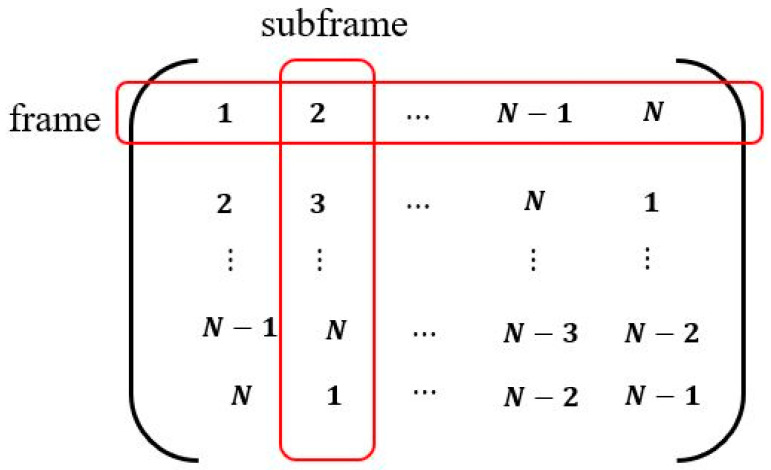
The data transmission matrix.

Before describing the scheduling of CUs, we first define the data transmission matrix ($M_{Data}$) which is an N-by-N matrix, as depicted in Figure 4. $m_{uv}$ is an element of $M_{Data}$ indexed by u for rows and v for columns where u and v are integers ranging from 1 to N, respectively. It is expressed as

(1) $$m_{uv}=\left\{\begin{array}{ccc} mod(u+v-1,\;N), & & mod(u+v-1,\;N)>0\\ N, & & mod(u+v-1,\;N)=0 \end{array}\right.$$

where $mod\left(x\right)$ is a modular function. For example, when N = 10, u = 2, and v = 5, $m_{uv}$ equals 6. Also, when N = 10, u=N, and v = 2, $m_{uv}$ equals 1. In this matrix, rows represent frame indices, and columns represent subframe indices, as shown in Figure 4. Thus, sender CUs can be determined for each subframe within a frame. Additionally, due to the symmetric nature of the matrix, CUs can have fair data transmission opportunities throughout N frames. This matrix is maintained by all CUs present in the network.

For a specific FI and SFI, the corresponding row and column indices in $M_{Data}$ are defined as $u_{tx}$ and $v_{tx}$, respectively. Using Equation (1) and the frame index FI, $u_{tx}$ is represented as

(2) $$u_{tx}=\left\{\begin{array}{ccc} mod(FI,\;N), & & mod(FI,\;N)>0\\ N, & & mod(FI,\;N)=0 \end{array}\right.$$

In addition, applying the subframe SFI into Equation (1), $v_{tx}$ is determined as

(3) $$v_{tx}=\left\{\begin{array}{ccc} mod(SFI,\;N), & & mod(SFI,\;N)>0\\ N, & & mod(SFI,\;N)=0 \end{array}\right.$$

Then, using Equations (2) and (3), the sender CU index ($i_{tx}$) for a specific FI and SFI is finally given as $i_{tx}=M_{Data}(u_{tx}{,v}_{tx})$. For example, when FI = 12 and SFI = 4, $u_{tx}$ and $v_{tx}$ are 2 and 4, respectively. Therefore, $i_{tx}$ = $M_{Data}$(2,4) = 5. This implies that the index of the sender CU in the fourth subframe of the twelfth frame is determined to be five.

The number of transmissions per frame for CUs is determined by the values of N and x. If x≤N (i.e., in the case that the number subframes is less than that of the CUs), then during one frame, CUs may either transmit once or not transmit at all. On the other hand, if x>N, CUs can be given more than one transmission opportunity within one frame. However, despite this, all CUs receive equal transmission opportunities over N frames. This case can be illustrated with an example where N = 10, x = 12, FI = 5, and SFI = 11. When FI = 5, $u_{tx}$ = 5, and when SFI = 11, $v_{tx}$ = 1. In this scenario, $i_{tx}$ = $M_{Data}$(5,1) = 5, which is consistent with the case when FI = 5 and SFI = 1. This indicates that even when x>N, the fifth CU is designed to have transmission opportunities in two subframes, SFI = [1, 11].

### 3.4. Data Channel Determination and Data Transmission Methods

Selecting the data channel index between sender and receiver CUs is based on their individual sensing results. Like MMAC-CR, we also employ ATIM and ATIM_ACK messages for this purpose in UMMAC-CA. The process of data channel determination is explained as follows:

- As depicted in Figure 3, the time allocated to (FI,SFI) consists of the time for exchanging ATIM-ATIM_ACK messages and the time for transmitting and receiving Data-Data_ACK messages. Here, each CU determines whether it is the Sender CU at (FI, SFI) based on the data transmission matrix as described in Section 3.3. The sender CU of the (FI,SFI) generates an ATIM message to the receiver CU, the destination where the sender CU tries to transmit its data. In this process, the ATIM message includes the indices of the sender CU index (i.e., $i_{tx}$) and the receiver CU index (i.e., $i_{rx}$), along with the status of each data channel sensed by the sender CU regarding the status of NCUs;
- The sender CU generates and transmits the ATIM message at the beginning of the subframe. This is because the sender CU may not know the current location of the receiver CU, so it needs to transmit the ATIM message within the maximum propagation delay time;
- The receiver CU verifies that the destination of the received ATIM message is itself. It then compares the channel sensing information received from the sender CU with its own channel sensing information. By excluding data channels where NCUs occur and randomly selecting a data channel from those where NCUs do not exist, the receiver CU determines the data channel (i.e., $c_{Data}$). This decision is communicated to the sender CU via an ATIM_ACK message, which includes the sender CU and receiver CU indices, the chosen data channel, and the receiver CU channel sensing information;
- The receiver CU generates and transmits the ATIM_ACK message immediately after the maximum propagation delay has elapsed since the start of the subframe. This is to ensure that the CUs located within the maximum propagation delay distance from the receiver CU can receive the ATIM_ACK message;

Next, we describe the data transmission and reception procedures between sender and receiver CUs in UMMAC-CA as follows:

- Upon receiving the ATIM_ACK message from the receiver CU, the sender CU confirms the selected data channel index and sets the transmission frequency accordingly. Subsequently, after a double maximum propagation delay (i.e., ${2\times \tau }_{p}$) from the start of the subframe, the sender CU transmits a data message to the receiver CU on the selected data channel. If the ATIM_ACK message is not received from the receiver CU by ${2\times \tau }_{p}$ from the start of the subframe, no data transmission occurs;
- The receiver CU that sent the ATIM_ACK message awaits data reception on the selected data channel. If the data message is received from the sender CU within ${(\delta +2)\times \tau }_{p}$ from the start of the subframe, the receiver CU generates and transmits an ACK message on that data channel. If no data is received until ${(\delta +2)\times \tau }_{p}$, no action is taken;
- Even if the sender CU receives or does not receive an ACK message from the receiver CU, it maintains its reception status in the next subframe since it did not perform a channel scan in this subframe. Additionally, the receiver CU also maintains its reception status in the next subframe.

In one subframe, the CUs other than the sender or receiver CUs maintain their reception status by default. Upon receiving ATIM or ATIM_ACK messages, they update the data channel sensing information sent by the respective sender CU and receiver CU. Additionally, during the exchange of Data-ACK messages between the sender CU and the receiver CU, they scan the data channels, thereby reducing the overall sensing time. Finally, they determine the mode of the next subframe based on the data transmission matrix (i.e., $M_{Data})$. That is, if the next subframe is allocated to them, they act as the sender CU; otherwise, they remain in the reception state.

## 4. Performance Analysis

In this section, we analyze the performance of UMMAC-CA and MMAC-CR for a distributed UCAN via simulations. To do this, we describe performance metrics, assumptions and conditions for simulations, and finally present the simulation results in sequence.

### 4.1. Performance Parameter Definitions

We consider the following four performance parameters in order to describe the superiority of UMMAC-CA over MMAC-CR:

- Sensing time ratio. This parameter is important because it shows whether enough time is guaranteed for detecting NCUs which randomly occur in time, space, and frequency domains;
- Controlling time ratio. This parameter is crucial as it indicates how much time a CU can allocate to data transmission rather than non-data communication;
- Throughput is a fundamental metric for evaluating the overall amount of data across the network, especially in challenging underwater communication environments prone to frequent communication failures;
- The message overhead is another critical network performance parameter. It assesses the number of messages generated for control and data transmission when applying UMMAC-CA, providing insights into protocol efficiency.

Considering the significance of the performance parameters, we define them one by one, in detail. First, the sensing time rate refers to the ratio of the time dedicated to sensing NCUs, occurring just before data transmission within a frame. The consideration of sensing time before data transmission is crucial because it allows for assessing the most recent statuses of NCUs occurring on a data channel, right before the data transmission takes place. The formulas of the sensing time rate of UMMAC-CA and MMAC-CR are individually determined as follows:

- In UMMAC-CA, during a frame, CUs can sense the status of NCUs during both the beacon subframe and the data communication between specific sender and receiver CUs within each subframe. In Figure 3, the overall sensing time in a frame is given as $\left[2+(\delta +1)\times x\right]\times \tau$. Therefore, the ratio of the sensing time to the frame length of UMMAC-CA is represented as $\frac{2+(\delta +1)\times x}{2+(\delta +3)\times x}$;
- In [25], it was stated that fast scanning occurs in Phase I and fine scanning occurs in Phase IV. However, sender and receiver CUs, aiming for data communication, have already completed data channel selection in Phase III. In Phase IV, they intend to transmit data using the DCF. Therefore, additional scanning in Phase IV does not influence the data communication between sender and receiver CUs. Furthermore, if the sensing information from the remaining CUs in Phase IV of the current frame is intended for use in that of the next frame, due to the long propagation delay of underwater acoustic signals, that sensing information is practically outdated. Consequently, in the UCAN environment where MMAC-CR is applied, CUs can effectively utilize only the sensing information from Phase I. In this regard, the sensing time rate of MMAC-CR is represented as $\frac{1}{2N+C+5}$.

Second, the controlling time rate is the ratio of time spent on non-data communication (beacon broadcasting, data channel selection, data channel sensing) to the length of a frame. A lower value of this parameter increases the time allocated for data communication within a frame, thereby enhancing network throughput. The equations of the controlling time rate of UMMAC-CA and MMAC-CR are given as follows:

- The controlling time of UMMAC-CA includes the time for beacon broadcasting and the exchange of ATIM-ATIM_ACK messages for data channel selection. Therefore, the controlling time is equal to 2×(x+1)×τ. Then, the ratio of the controlling time to the frame length of UMMAC-CA is determined as $\frac{2\times (x+1)}{2+(\delta +3)\times x}$;
- In MMAC-CR, all phases except Phase IV are included in the controlling time. Hence, the controlling time rate of MMAC-CR is expressed as $\frac{2N+C+1}{2N+C+5}$.

Third, throughput is defined as the ratio of the amount of data successfully transmitted in a frame to the maximum amount of data that can be transmitted in the same frame. The maximum amount of data refers to the quantity of data that can be transmitted between sender and receiver CUs in each subframe without any collisions from NCUs. The equations of the throughputs of UMMAC-CA and MMAC-CR are defined as follows:

- In UMMAC-CA, when there is no collision from NCUs during one frame and the same amount of data is transmitted during each subframe, the total amount of data is represented by $x\times \delta \times DR\times \tau_{d}$. However, collisions from NCUs can occur during each subframe. Considering this, the total amount of data, accounting for such collisions, is represented by $DR\times \delta \times \tau_{d}\times \sum_{SFI=1}^{x}p_{SFI}$. Thus, the throughput of UMMAC-CA is represented as $\frac{\sum_{SFI=1}^{x}p_{SFI}}{x}$;
- In MMAC-CR, the maximum amount of data without collisions from NCUs is represented by $DR\times C_{AC}\times \tau_{d}$, while the data amount considering collisions is represented by $DR\times \tau_{d}\times \sum_{l=1}^{C_{AC}}p_{l}$. Thus, the throughput of MMAC-CR is represented as $\frac{\sum_{l=1}^{C_{AC}}p_{l}}{C_{AC}}$.

Fourth, the message overhead refers to the number of messages generated within a single frame. When deriving the message overhead, the transmission of messages depends on the channel state, leading to countless scenarios. Therefore, considering no errors due to poor channels, we calculate the maximum message overhead that can occur within a single frame. In addition, we consider a scenario where a single CU successfully broadcasts a beacon message without collisions in both UMMAC-CA and MMAC-CR. Beacon broadcasting is performed commonly in both MAC protocols, so it does not affect the comparison of message overhead performance. Therefore, this simple assumption poses no issue when comparing the message overhead performance of the two MAC protocols under fair conditions. Then, the formulas of the message overheads of UMMAC-CA and MMAC-CR are, respectively, determined as follows:

- In UMMAC-CR, there is only one message generated due to beacon broadcasting, while the number of messages resulting from ATIM-ATIM_ACK exchanges is 2×x considering the number of subframes. And the number of messages generated by Data-ACK messages is $\sum_{SFI=1}^{x}\left(1+p_{SFI}\right)$ depending on the occurrence of collisions with NCUs. Thus, the overall message overhead of UMMAC-CA is given as $1+2\times x+\sum_{SFI=1}^{x}\left(1+p_{SFI}\right)$;
- In MMAC-CR, during Phase I, one broadcasting message is generated, during Phase II, there are as many data channel-sensing-related messages as the number of data channels (i.e., C). During Phase III, the number of ATIM-ATIM_ACK messages generated is equal to the number of CUs intending to send data in one frame (i.e., $2\times N_{D}$). In Phase IV, the number of messages can vary depending on the presence of collisions with NCUs, affecting the number of RTS, CTS, Data, and ACK messages. Therefore, the number of messages in Phase IV can be represented as $\sum_{l=1}^{C_{AC}}O_{l}$. If RTS transmission fails due to collisions with NCUs, $O_{l}$ is 1. When only RTS transmission is successful, $O_{l}$ is 2. Furthermore, if RTS and CTS transmissions are successful, $O_{l}$ is 3, and if RTS, CTS, and Data are successfully transmitted, $O_{l}$ is 4. The total message overhead for a frame is $2\times N_{D}+C+1+\sum_{l=1}^{C_{AC}}O_{l}$.

Finally, all equations of the sensing time rates, controlling time rates, throughputs, and message overheads for UMMAC-CA and MMAC-CR are defined in Table 2.

### 4.2. Assumptions and Conditions for Simulations

Since underwater acoustic communication operates in an open spectrum, there is currently no standardized frequency system and protocol definition for different layers. Moreover, research on networking for UCANs is still in its early stages. In such an environment, we have been developing standardized frequency schemes and channel sharing protocols for UCANs, as in [7,13,15]. Therefore, in the simulations, we apply the assumptions used in [7,13,15] as follows:

- All CUs exist within the network (i.e., they can communicate within one hop), and the entry of new CUs into the network is not considered in this study;
- All CUs are equipped with a CA-enabled communication module that allows for the scanning and tuning of control and data channels;
- To purely compare the performance of two MAC protocols, all messages are transmitted successfully without loss due to harsh channel environments;
- For a fair comparison with UMMAC-CR, in MMAC-CR, in Phase III, all CUs perform data channel selection without collisions. Moreover, in Phase IV, only data communication between specific sender and receiver CUs occurs on available data channels;
- While CUs are mobile, they do not leave the network coverage area;
- The occurrence locations, times, and durations of NCUs are random, and NCUs occurring within the maximum communication range of CUs can be sensed;
- All CUs are fully occupied, and the buffer capacity is sufficient to prevent data loss.

The best way to testify the performance of UMMAC-CA is to implement the protocol and apply it underwater. However, prior to underwater validation, simulations are also necessary to confirm the functionality and performance of UMMAC-CA. Although the simulations considered in this study, including the 3D network structure, propagation delay, transmission delay, varying numbers of CUs, and the irregular occurrence of NCUs per data channel, do not perfectly replicate real underwater environments, they are set up to at least investigate whether UMMAC-CA can be used in underwater environments. The simulation is conducted using MATLAB software under the following conditions:

- We consider a three-dimensional network topology as shown in Figure 2, as well as the time and frequency domain fragmentation as depicted in Figure 3;
- The sea depth (d) and the maximum communication range (R) are given as 100 and 5000 m, respectively;
- The moving velocity of a CU is given as 2 mps;
- The data rate (DR) is 1 kbps;
- The transmission delay ($\tau_{d}$) is set to 0.5 s;
- The maximum propagation delay is given as 3.33 s considering the average acoustic speed is 1500 mps and the maximum communication range of 5000 m;
- The sum of the maximum propagation delay, transmission delay, and gourd time are set to 4 s (i.e., τ);
- The data transmission time coefficient (δ) is given as 1:1:10;
- The number of data channels (C) is 25;
- The number of CUs is given as the set of [10, 25, 50]. This is set to account for scenarios where the number of CUs is smaller than, equal to, or greater than the number of data channels;
- $T_{MAX}$ is set equal to T, implying that the occurrence time duration of an NCU is uniformly distributed in the range of [1, *T*]. The occurrence time of an NCU ($t_{NCU}$) is also uniformly distributed in the range of [1, *T*]. From these conditions, an NCU can exist over two frames at most;
- The number of NCUs that occur at one data channel per frame follows a Poisson distribution with an average number of NCUs per frame, $\lambda_{NCU}$, varying from 1.0 to 5.0 in steps of 2.0;
- The simulation time, expressed as the number of total frames ($N_{SIM}$), is set to $10^{6}$.

In addition, the following two simulation scenarios are considered in order to investigate the effect of simulation conditions (i.e., $\lambda_{NCU}$, $N_{CU}$, and δ):

- Analyzing the performance by fixing the average number of NCUs ($\lambda_{NCU}$) occurring on a single channel and varying the data transmission time coefficient (δ) and the number of CUs (N);
- Investigating the performance by fixing the number of CUs (N) and varying the data transmission time coefficient (δ) and the average number of NCUs occurring on a single channel ($\lambda_{NCU}$);
- In each scenario, $10^{6}$ simulations are executed for each condition, and the average performance is obtained.

### 4.3. Simulation Results

**Figure 5:**
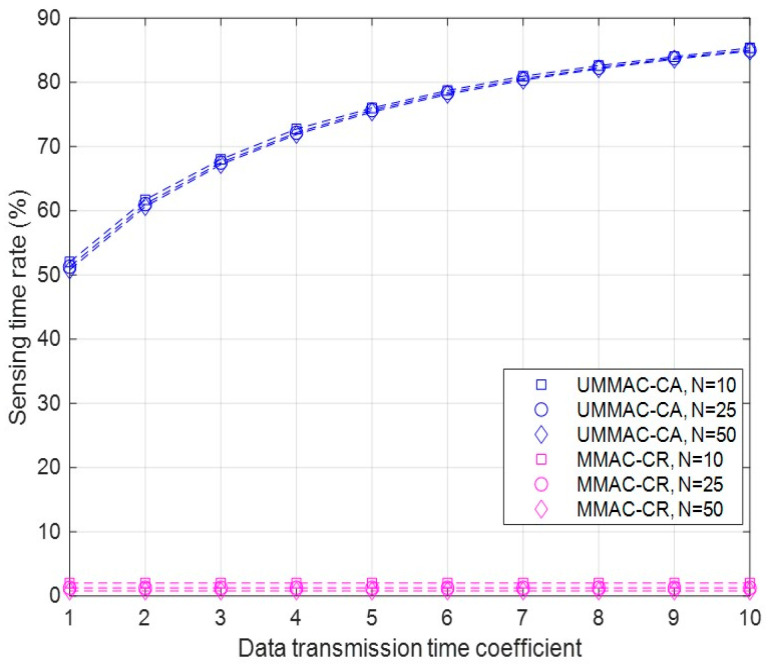
The sensing time rate according to the data transmission time coefficient and the number of CUs.

First, the results of the sensing time rate of UMMAC-CA and MMAC-CR are outlined as follows:

- As defined in the sensing time rate, sufficient sensing time must be ensured to adequately detect NCUs before data transmission can occur. Since the sensing time rate is independent from NCU occurrences, simulations are conducted varying only the number of CUs and the data transmission time coefficient to derive the test results;
- As depicted in Figure 5, simulation results show that UMMAC-CR guarantees at least 50% of the sensing time rate regardless of the variable conditions. As the data transmission time coefficient increases, the number of subframes decreases. Consequently, the length of data transmission increases.

- This time represents the duration of data transmission for sender and receiver CUs, but for other CUs, it becomes the time for data channel sensing. Therefore, a longer data transmission time allows for more sensing;
- In contrast, MMAC-CR achieves a sensing time rate of only 2% of the total frame. This is because the sensing results in Phase I can be only used in data transmission in Phase IV. A short sensing time implies unreliable results for sensing before data transmission, which can be critical for network performance, especially with greater randomness in NCU occurrences;
- Regarding the number of CUs, while the sensing time rate decreases as the number of CUs increases in MMAC-CR, the difference is unremarkable. For UMMAC-CR, as the number of CUs increases, the number of subframes increases, leading to an increase in both data transmission and sensing time, resulting in almost no difference in the sensing time rate, as shown in Figure 5. Therefore, the number of CUs does not significantly affect the sensing time rate, indicating that both media access control mechanisms provide a consistent sensing time rate regardless of the number of CUs;
- As a result, UMMAC-CA can significantly increase the sensing time rate compared to MMAC-CR, but it may consume more received power for sensing the data channels accordingly. While in MMAC-CR, all CUs only sense the data channels in Phase I, in UMMAC-CA, N−2 CUs not involved in data transmission in each subframe sense the data channels. Let us denote the received power per data channel as $P_{rx}$. The total power consumption for receiving for the N−2 CUs not transmitting data in one subframe is $(N-2)\times C\times P_{rx}$, where C is the number of data channels. Therefore, as one frame consists of x subframes, an additional received power consumption of $x\times (N-2)\times C\times P_{rx}$ is incurred. This additional power consumption can be considered as the trade-off for UMMAC-CA ensuring a 50% sensing time compared to MMAC-CR.

**Figure 6:**
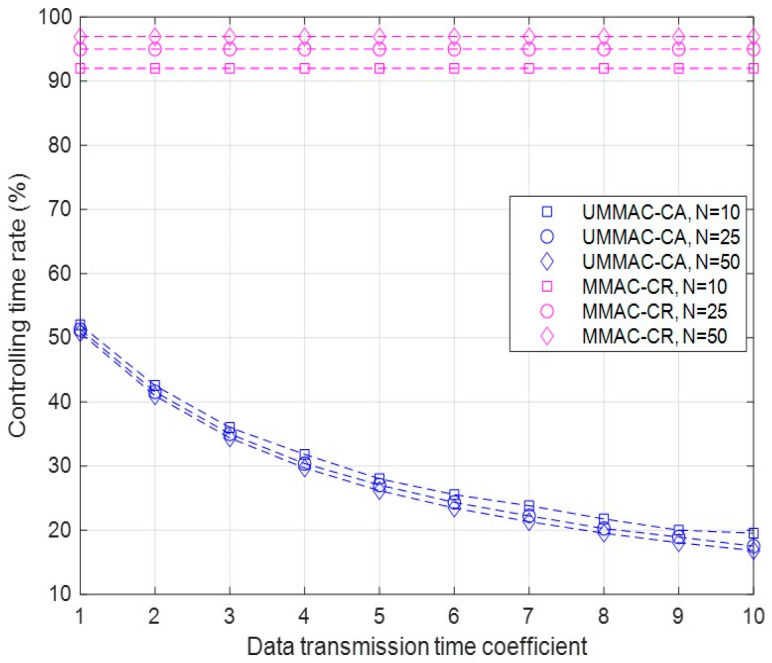
The controlling time rate according to the data transmission time coefficient and the number of CUs.

Second, the results of the controlling time rate of UMMAC-CA and MMAC-CR are described as follows:

- The controlling time rate represents the proportion of time allocated to non-data transmission relative to the frame, indicating how long non-data communication time is incurred through non-data transmission to send data. Since the controlling time rate is also independent from NCU occurrences, the results are derived by varying only the number of CUs and the data transmission time coefficient;
- In the case of MMAC-CR, the time excluding Phase IV, where data transmission occurs, corresponds to the time related to non-data communication. In the case of UMMAC-CR, this includes the beacon transmission and data channel selection time;
- Simulation results show that regardless of variable conditions, UMMAC-CR exhibits significantly lower controlling time rates compared to those of MMAC-CR, as shown in Figure 6. This is because in UMMAC-CR, while specific sender and receiver CUs engage in data transmission, the rest of the CUs engage in channel sensing, sharply reducing channel sensing time. Furthermore, for data transmission time coefficients above 2.0, UMMAC-CR reduces the controlling time rate by over 50% compared to that of MMAC-CR;

- In UMMAC-CR, there is an almost inverse relationship between the sensing time rate performance and the controlling time rate performance. This is because, excluding beacon broadcasts, the sensing time directly corresponds to the data transmission time between specific sender and receiver CUs, while the non-data communication time represents the time excluding data transmission time in the entire frame. Therefore, the pattern of controlling time rate performance based on the number of CUs and the data transmission time is opposite to that of the sensing time rate performance, as shown in Figure 5 and Figure 6. On the other hand, in MMAC-CR, since the sensing time is part of the non-data communication time, the controlling time rate is much larger than the sensing time rate, as depicted in Figure 5 and Figure 6.

**Figure 7:**
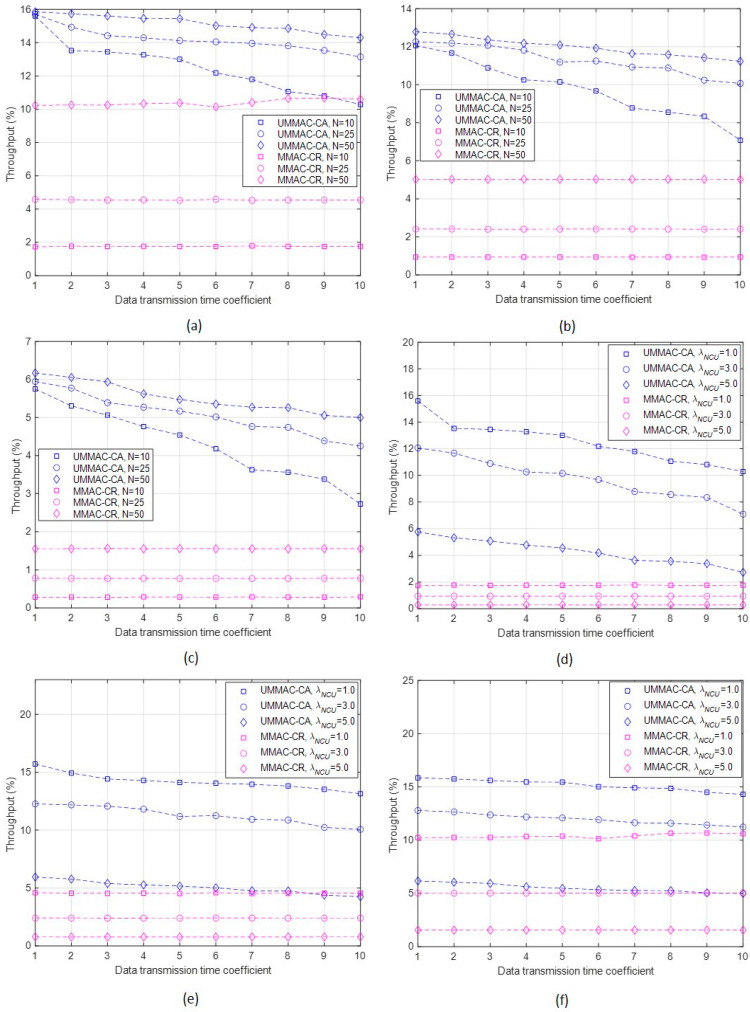
Throughput according to δ, $\lambda_{NCU}$, and N. (**a**) Throughput according to δ and N when $\lambda_{NCU}$ = 1.0. (**b**) Throughput according to δ and N when $\lambda_{NCU}$ = 3.0. (**c**) Throughput according to δ and N when $\lambda_{NCU}$ = 5.0. (**d**) Throughput according to δ and $\lambda_{NCU}$ when N=10. (**e**) Throughput according to δ and $\lambda_{NCU}$ when N=25. (**f**) Throughput according to δ and $\lambda_{NCU}$ when N=50.

Third, the results of the throughputs of UMMAC-CA and MMAC-CR are summarized as follows:

- The throughput performance represents the ratio of the total amount of data transmitted on non-collision data channels to the amount of data that would be transmitted when all channels are utilized (i.e., in the NCU collision-free case). This performance metric allows us to assess how much data transmission occurred on idle data channels by avoiding collisions from NCUs;
- In the case of UMMAC-CA, regardless of the conditions of the average NCU occurrence per data channel (i.e., $\lambda_{NCU}$) and the number of CUs (N), the throughput decreases with the data transmission time coefficient value (δ), as depicted in Figure 7a–f. As the data transmission time coefficient increases, the sensing time also increases (confirmed through the sensing time rate results). However, higher data transmission times can also increase the probability of collision with undetected NCUs, reducing the probability of successful data transmission during this time. To improve throughput in UMMAC-CA, it is necessary to reduce the data transmission time. This can be achieved by shortening the sensing time in one subframe to decrease the probability of interference with NCUs;
- In contrast, MMAC-CR shows throughput performance independent of the data transmission time coefficient. This can be inferred from the definition of throughput and confirmed through simulations, as shown in Figure 7. In addition, reducing the data transmission time coefficient in UMMAC-CA can mitigate the difference in throughput between UMMAC-CA and MMAC-CR. However, through simulations under the same conditions (the number of CUs and the number of NCUs), UMMAC-CA consistently outperforms MMAC-CR in terms of throughput;
- Throughput performance increases with a larger number of CUs in both MAC protocols. In the case of UMMAC-CA, as the number of CUs increases, the number of subframes in one frame (x) increases. This results in an increase in both the numerator and denominator of the throughput formula, but the increase in x enables more data transmission, leading to an overall increase in throughput, as illustrated in Figure 7a–c. For MMAC-CR, an increase in CUs increases the probability of using idle data channels, ultimately leading to an increase in throughput. Since both MAC protocols operate on a non-contention basis, the increased competition with an increase in the number of CUs has less impact on throughput performance compared to the greater likelihood of channels being ‘under-utilized’ when the CU count is low;
- Conversely, throughput increases as the number of NCUs decreases (i.e., when $\lambda_{NCU}$ is low) for both MAC protocols, as shown in Figure 7d–f. This is because a lower probability of NCU occurrences reduces the likelihood of collisions with NCUs, leading to an increased probability of successful data transmission and, thus, enhanced throughput performance;
- Comparatively, regardless of the values of $\lambda_{NCU}$ and N, UMMAC-CA outperforms MMAC-CR in throughput. This is because UMMAC-CA periodically executes sensing and data transmission through multiple subframes. The performance difference is more remarkable with lower data transmission time coefficients, smaller numbers of CUs, and smaller numbers of NCUs. For instance, when δ is 2.0, UMMAC-CA exhibits a throughput performance improvement ranging from a minimum of 37% to a maximum of 90%.

**Figure 8:**
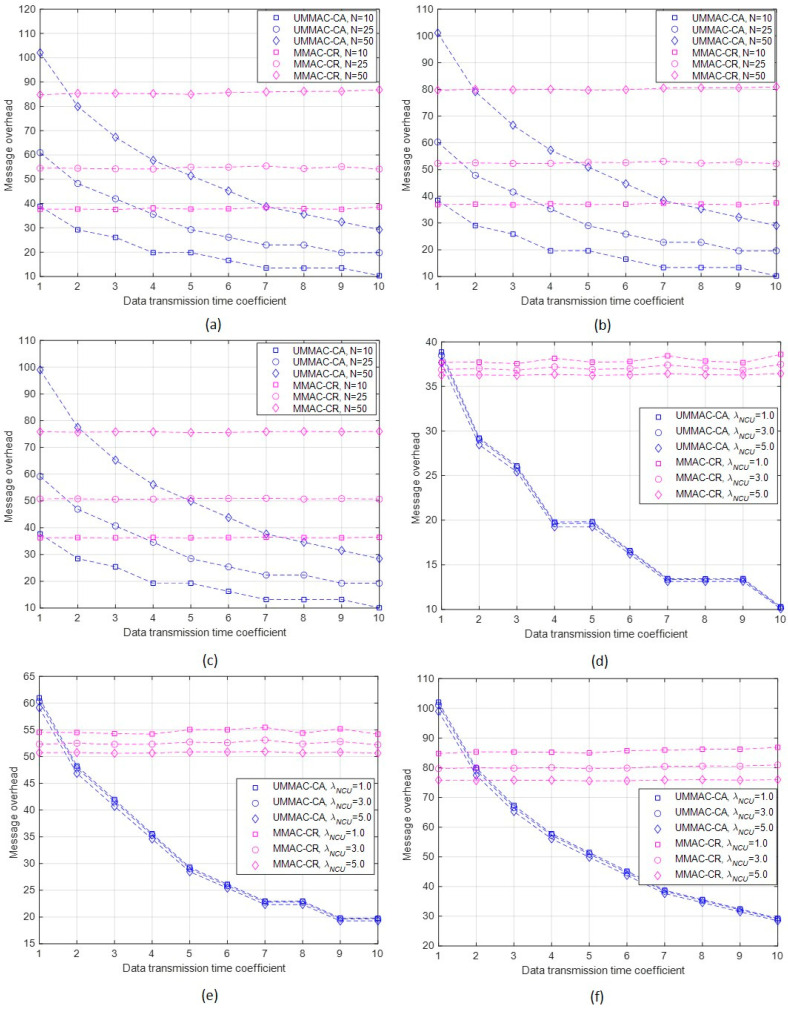
Message overhead according to δ, $\lambda_{NCU}$, and N. (**a**) Message overhead according to δ and N when $\lambda_{NCU}$ = 1.0. (**b**) Message overhead according to δ and N when $\lambda_{NCU}$ = 3.0. (**c**) Message overhead according to δ and N when $\lambda_{NCU}$ = 5.0. (**d**) Message overhead according to δ and $\lambda_{NCU}$ when N=10. (**e**) Message overhead according to δ and $\lambda_{NCU}$ when N=25. (**f**) Message overhead according to δ and $\lambda_{NCU}$ when N=50.

Fourth, the results of the message overheads of UMMAC-CA and MMAC-CR are detailed as follows:

- The message overhead performance refers to the number of messages generated during the execution of a single frame. A higher message overhead can lead to increased resource consumptions such as frequency usage, transmission time, and power consumption;
- In the case of UMMAC-CA, regardless of the values of $\lambda_{NCU}$ and N, the message overhead decreases with the increase in δ, as illustrated in Figure 8a–f. This is because as δ increases, the number of subframes decreases, causing a reduction in the exchange of ATIM-ATIM_ACK messages for data channel selection. Additionally, as δ increases, the exchange of Data-ACK messages also decreases, resulting in an overall reduction in the message overhead. Conversely, MMAC-CR shows results independent from the δ values. This is evident from the definition of message overhead and confirmed through simulations. When δ equals 1, it corresponds to the maximum number of subframes, and this is the only scenario where the message overhead performance of MMAC-CR surpasses that of UMMAC-CA. Except for this case, under given conditions, UMMAC-CA outperforms MMAC-CR in terms of message overhead performance;
- The message overhead performance of both MAC protocols increases with an increase in the number of CUs, as shown in Figure 8a–c. For UMMAC-CA, a higher N value leads to an increase in the number of subframes, thereby increasing the message overhead. This contradicts the inverse relationship between message overhead performance and δ values. For MMAC-CR, the increase in the number of CUs results in an increase in message overhead across all phases except Phase I;
- The message overhead performance increases with a decrease in the number of NCUs (i.e., smaller $\lambda_{NCU}$ values) for both MAC protocols, as depicted in Figure 8d–f. This is because a lower probability of NCU occurrence increases the likelihood of successful data transmission, consequently increasing the number of messages. However, the impact of NCUs primarily affects the exchange of Data-ACK messages. This influence is less significant on message overhead compared to the number of CUs, which affects all messages generated in the frame;
- Comparatively, for δ values greater than or equal to 2.0, UMMAC-CA exhibits a lower message overhead compared to MMAC-CR regardless of $\lambda_{NCU}$ and N conditions. This is because MMAC-CR consistently generates a significant number of control messages, except in Phase IV, whereas the message generation in UMMAC-CA depends on the number of subframes. Therefore, as δ increases (i.e., as the number of subframes decreases) or as the number of CUs increases, the performance difference in message overhead becomes more remarkable.

Finally, we outline the overall simulation results as shown in Table 3. Summarizing the simulation results, UMMAC-CA has been designed to be adaptive based on the δ value, ensuring that it can sense for at least 50% of the time within a frame at the expense of increasing received power consumption. This implies a reduction in the proportion of non-data transmission time. Furthermore, UMMAC-CA outperforms MMAC-CR in terms of sensing time ratio, controlling time ratio, and throughput, through the enhanced sensing of more NCUs. Except for the case where δ equals 1, the message overhead performance of UMMAC-CA is also superior to that of MMAC-CR. This indicates that UMMAC-CA is well-designed to complement MMAC-CR in underwater environments. In addition, regarding the setting of the δ value in our UMMAC-CA, the following points can be summarized:

- UMMAC-CA is designed to adjust its performance metrics by tuning the value of δ for data transmission time, in comparison to MMAC-CR. It has been observed that the performance metrics, except for message overhead, are inversely related to the delta value. In other words, lower δ values cause better throughput performance but deteriorate other performance metrics;
- With δ = 1.0, throughput can achieve its maximum irrespective of the network size, but performance varies significantly concerning the number of CUs and NCUs for higher δ values. Except for message overhead performance, UMMAC-CA demonstrates superiority over MMAC-CR for most performance metrics when δ equals 1.0;
- In severe underwater conditions, setting δ to 1.0 seems preferable as it guarantees stable throughput performance even with slightly increased message overhead.

## 5. Conclusions

The cognitive communication in a distributed underwater acoustic network can be challengeable due to the absence of sharing network-wide sensing information provided by a central entity. This stems mainly from frequent collisions with NCUs and neighboring CUs, especially in harsh channel conditions. However, there are numerous underwater applications that require a distributed network topology without centralized management. In such cases, a distributed UCAN is essential, necessitating a tailored MAC protocol designed specifically for this network architecture.

In this paper, we proposed a MAC protocol referred to as UMMAC-CA, based on MMAC-CR targeted for terrestrial distributed CRNs. UMMAC-CA performs sequential sensing, data channel selection, and data transmission on a per-frame basis, similar to MMAC-CR. Unlike MMAC-CR, UMMAC-CA adeptly handles the random occurrence of NCUs by dividing a single frame into multiple subframes. In addition, the data transmission sequence per subframe is determined by a pre-defined scheduling referred to as the data transmission matrix. Through the data transmission matrix, all CUs can access any data channel periodically and fairly across frames. This matrix also allows one pair of CUs to perform data transmission in a subframe while enabling the remaining CUs to sense for NCUs in all data channels in that subframe. Hence, UMMAC-CA not only removes the overhead linked with channel access but also guarantees that at least half of a frame is dedicated to sensing. This can be crucial for detecting randomly appearing NCUs to prevent collisions with them.

Simulation results showed that UMMAC-CA outperforms MMAC-CR in terms of sensing time rate, controlling time rate, and throughput, regardless of simulation conditions. In addition, except for the case where the data transmission time coefficient equals 1, the message overhead performance of UMMAC-CA is also superior to that of MMAC-CR. This implies that UMMAC-CA is well-designed to complement MMAC-CR in underwater environments. Particularly, it reduces the controlling time by at least 50% and improves throughput by at least 37%. Moreover, it was observed that UMMAC-CA provides the highest throughput when the data transmission time coefficient is set to 1.0. Based on the simulation results, it can be concluded that UMMAC-CA can be widely applied to underwater applications capable of multi-channel cognitive communication within a distributed network structure.

## Figures and Tables

**Table 1:** The definition of parameters to describe UMMAC-CA.

Parameters	Description
i	CU index
$i_{tx}$	Sender CU index
$i_{rx}$	Receiver CU index
$c_{Data}$	Data channel index reserved between $i_{tx}$ and $i_{rx}$
δ	Data transmission time coefficient
x	The number of subframes
FI	Frame index
SFI	Subframe index
T	The length of a frame
DR	Data rate
$p_{SFI}$	The collision probability caused by NCUs in one subframe of UMMAC-CR (0: collision, 1: no collision)
$M_{AC}$	The set of data channels not sensed by NCUs during a frame of MMAC-CR
$C_{AC}$	$\mathrm{The}\;\mathrm{size}\;\mathrm{of}\;M_{AC}$$\;(=\left|M_{AC}\right|$)
l	The index of data channels belonging to $M_{AC}$
$p_{l}$	$\mathrm{The}\;\mathrm{collision}\;\mathrm{probability}\;\mathrm{of}\;\mathrm{NCUs}\;\mathrm{in}\;\mathrm{data}\;\mathrm{channel}\;M_{AC}(l)$ (0: collision, 1: no collision)
$N_{D}$	The number of CUs with data to transmit in one frame in MMAC-CR
R	The maximum communication range
d	The sea depth

**Table 2:** The performance parameters of UMMAC-CA and MMAC-CR.

Parameters	UMMAC-CA	MMAC-CR
Sensing time rate	$\frac{2+(\delta +1)\times x}{2+(\delta +3)\times x}$	$\frac{1}{2N+C+5}$
Controlling time rate	$\frac{2\times (x+1)}{2+(\delta +3)\times x}$	$\frac{2N+C+1}{2N+C+5}$
Throughput	$\frac{\sum_{SFI=1}^{x}p_{SFI}}{x}$	$\frac{\sum_{l=1}^{C_{AC}}p_{l}}{C_{AC}}$
Message overhead	$1+2\times x+\sum_{SFI=1}^{x}\left(1+p_{SFI}\right)$	$2\times N_{D}+C+1+\sum_{l=1}^{C_{AC}}O_{l}$

**Table 3:** Summary of simulation results.

Parameters	Conditions		UMMAC-CA		MMAC-CR	
			Min	Max	Min	Max
Sensing time rate (%)	N=10		52.0	85.3	2.0	2.0
	N=25		51.2	85.0	1.3	1.3
	N=50		50.7	84.8	0.7	0.7
Controlling time rate (%)	N=10		19.5	52.0	92.0	92.0
	N=25		17.5	51.2	95.0	95.0
	N=50		16.8	50.7	96.7	96.7
Throughput (%)	N=25	$\lambda_{NCU}$ = 1.0	13.1	15.7	4.5	4.5
		$\lambda_{NCU}$ = 3.0	10.1	12.3	2.4	2.4
		$\lambda_{NCU}$ = 5.0	4.3	5.9	0.8	0.8
	$\lambda_{NCU}$ = 3.0	N=10	7.1	12.1	0.9	0.9
		N=25	10.1	12.3	2.4	2.4
		N=50	11.2	12.8	5.0	5.0
Message overhead	N=25	$\lambda_{NCU}$ = 1.0	19.8	61.0	54.2	54.5
		$\lambda_{NCU}$ = 3.0	19.6	60.3	52.2	52.3
		$\lambda_{NCU}$ = 5.0	19.3	59.1	50.6	50.7
	$\lambda_{NCU}$ = 3.0	N=10	10.2	38.5	36.9	37.5
		N=25	19.6	60.3	52.2	52.3
		N=50	29.0	101.1	79.7	80.95

## Data Availability

The original contributions presented in the study are included in the article, further inquiries can be directed to the corresponding author.
